# Developing a comprehensive scale for measuring explanatory models of mental disorders among Japanese adults

**DOI:** 10.3389/fpsyg.2026.1834088

**Published:** 2026-07-29

**Authors:** Time Fukuda, Daisuke Kobayashi, Koubun Wakashima

**Affiliations:** 1 Graduate School of Education, Tohoku University, Sendai, Japan; 2 Graduate School of Clinical Psychology, Niigata Seiryo University, Niigata, Japan

**Keywords:** attribution of responsibility, explanatory model, health locus of control, mental disorder, vignette

## Abstract

**Introduction:**

Individuals differ in their understanding of the causes of illnesses or problems and the appropriate methods for addressing them. Mental disorders can be interpreted from multiple perspectives shaped by cultural backgrounds and personal experiences. The conceptual framework through which individuals recognize and interpret illnesses or problems is referred to as an explanatory model. This study aimed to develop the Explanatory Model Scale for Mental Disorders (EMS-MD), based on Haslam's (2003) explanatory styles-“Medicalizing,” “Moralizing,” and “Psychologizing”-with the addition of “Socializing” and “Spiritualizing,” and to examine its reliability and validity among Japanese adults.

**Methods:**

An online survey was administered to 928 Japanese adults aged 18 years and older using five mental disorder vignettes (depression, schizophrenia, alcohol use disorder, anorexia nervosa, and social anxiety disorder). A split-sample approach was used to conduct exploratory and confirmatory factor analyses. Internal consistency, test-retest reliability, convergent and discriminant validity, and measurement invariance across gender and vignette contexts were also examined.

**Results:**

The analyses suggested a 24-item, 6-factor structure comprising “Socializing,” “Spiritualizing,” “Biologizing,” “Medicalizing,” “Moralizing,” and “Psychologizing.” Notably, “Biologizing” and “Medicalizing” emerged as distinct factors, although this distinction was exploratory because both had originally been conceptualized as part of a broader “Medicalizing” category. Most subscales demonstrated adequate internal consistency, and all exhibited good test-retest reliability. However, “Psychologizing” showed low internal consistency, indicating that this subscale should be interpreted cautiously and requires further item refinement of its item pool. Correlations with measures of the Attribution of Responsibility Process and the Japanese Health Locus of Control subscales were generally consistent with predictions, providing preliminary evidence for convergent validity. The Fornell-Larcker criterion provided preliminary evidence for discriminant validity. Multigroup analyses indicated that the measurement structure was generally stable across gender; however, measurement invariance across vignette contexts was limited.

**Discussion:**

Overall, the EMS-MD shows promise as a preliminary measure of explanatory styles for mental disorders among Japanese adults. Nevertheless, limitations related to model fit, the internal consistency of “Psychologizing,” and measurement equivalence across vignettes highlight the need for further item refinement and validation in other cultural contexts.

## 1 Introduction

Globally, psychiatric care has shifted from hospital-based care to community-based care (World Health Organization, 2022). In Japan, efforts have similarly focused on creating an inclusive community society (Ministry of Health, Labour and Welfare, 2022). For people with mental disorders to continue living in the community, understanding and acceptance from family members, the general public, and healthcare professionals are essential. Mental health literacy has been widely promoted to enhance public understanding of mental disorders by disseminating accurate information (Jorm, 2000). However, presenting expert knowledge as the sole “correct” perspective does not fully capture how laypeople actually interpret and explain mental disorders. Moreover, biomedical explanations do not necessarily reduce stigma and may even increase perceptions of dangerousness and social distance (Kvaale et al., 2013).

In this context, Kleinman (1980) argued that clinicians, patients, family members, and communities each have their own explanatory models of illness and treatment. These models encompass beliefs about how illnesses develop and how they should be addressed. In community settings, the explanatory frameworks used by people with mental disorders and those around them may influence attitudes and helping behaviors. Therefore, understanding how adults interpret specific manifestations of mental disorders is important for examining public perceptions of mental illness.

Research on explanatory models has primarily relied on qualitative, semi-structured interviews, including the Explanatory Model Interview Catalogue (Weiss, 1997), the Short Explanatory Model Interview (Lloyd et al., 1998), and the McGill Illness Narrative Interview (Groleau et al., 2006). Although these methods are valuable for exploring illness experiences within cultural contexts, they are less suitable for large-scale quantitative research. Existing measures, such as the Barts Explanatory Model Inventory (Rüdell et al., 2009) and the IPQ-MH (Witteman et al., 2011), primarily assess structural dimensions of illness representations, including causes, course, consequences, and treatment. Consequently, they are less effective in capturing the explanatory styles through which mental disorders are understood across these domains. Therefore, a scale that quantitatively assesses multiple explanatory styles is needed.

This study is based on Haslam's (2003) classification of explanatory styles as its theoretical foundation. Haslam identified three explanatory styles that laypeople use to understand mental disorders and problematic behaviors: “Medicalizing,” “Moralizing,” and “Psychologizing.” “Medicalizing” frames a problem as a biomedical abnormality; “Moralizing” attributes it to character, willpower, or self-control; and “Psychologizing” explains it in terms of psychological stress or processes. Although this framework provides a useful foundation, explanations of mental disorders are not limited to biological, moral, or psychological processes within the individual. Prior research has also identified explanations based on social factors, such as poverty, discrimination, and economic instability (Aidoo and Harpham, 2001; Choudhry et al., 2016), as well as spiritual explanations involving divine punishment, curses, or other spiritual influences (Lloyd et al., 1998; McCabe and Priebe, 2004; Bhikha et al., 2012). Recent research has highlighted the importance of incorporating biological, psychological, social, and spiritual dimensions into explanatory frameworks (Dumke et al., 2023). Accordingly, in addition to Haslam's three styles, this study conceptualized explanations based on social factors as “Socializing” and those based on supernatural or religious factors as “Spiritualizing,” resulting in five hypothesized explanatory styles. The conceptual definitions of these styles are presented in Table 1.

**Table 1:** Conceptual definitions of explanatory styles.

Medicalizing	A tendency to attribute etiology to biomedical abnormalities or defects, perceiving behaviors and circumstances associated with the disorder as unintentional.
Moralizing	A tendency to attribute etiology to individual responsibility as a deviation from moral and social norms, perceiving behaviors and circumstances associated with the disorder as intentional, stemming from issues with personal character or self-control.
Psychologizing	A tendency to explain the disorder in relation to psychological factors such as stress—while acknowledging the difficulty in identifying a specific cause—and to perceive associated behaviors and circumstances as unintentional.
Socializing	A tendency to attribute etiology to social factors, perceiving systemic deficiencies or structural flaws as the core problem.
Spiritualizing	A tendency to explain the disorder in relation to religious, spiritual, or supernatural factors, perceiving their mechanisms or influences as the core problem.

To verify the developed scale's convergent validity, the “Attribution of Responsibility” (Weiner, 1995) and “Health Locus of Control” (HLC) frameworks were employed. According to Weiner's (1995) framework, cognitive appraisals regarding the cause of an event—such as whether it is “internal or external to the individual (locus of causality),” “stable (stability),” “controllable (controllability),” “severe (severity),” “responsible for the onset,” and “responsible for recovery (responsibility)”—determine emotional reactions (anger, sympathy) and behavioral reactions (helping behavior). For example, if the cause of a mental disorder is perceived to lie within an individual's character, the responsibility for both onset and recovery is often attributed to the person in question, making it difficult to garner sympathy. As the explanatory styles measured in this study inherently encompass judgments concerning the “internal-external locus” and “controllability” of etiology, they are predicted to correlate with cognitive appraisals, as well as emotional and behavioral reactions within the attribution of responsibility process.

HLC is a concept that applies Rotter's (1966) Locus of Control to the health domain, referring to the belief that health and illness are determined by personal actions, luck, or external factors, such as medical professionals. In Japan, Horike (1991) identified five factors within the Japanese HLC structure: “Supernatural” (seeking solutions from deities or ancestors), “Internal” (attributing causes to oneself), “Chance” (attributing causes to fate), “Family” (seeking solutions from close relatives), and “Professional” (seeking solutions from medical practitioners). Given that explanatory models of mental disorders involve assumptions about “what dictates the occurrence and recovery from an illness,” they are theoretically presumed to correspond with each dimension of the HLC. The hypotheses regarding each explanatory style are described below.

First, “Medicalizing”—a style attributing etiology to biological factors—is predicted to show a positive correlation with “Chance”—the belief that health is dictated by fate or coincidence—because biological predispositions are perceived as innate or inescapably given. It is also expected to correlate positively with “Professional,” reflecting a heightened desire for medical treatment. Furthermore, because disorders caused by biological factors are often perceived as difficult for the individual to control and require expert intervention, it is hypothesized to correlate negatively with “controllability” and positively with “severity.”

Second, “Moralizing” treats personal responsibility as a matter of individual character or morality. Consequently, it is predicted to align with the “internal attribution” of causes, and correlate positively with “controllability.” Such perceptions are linked to judgments that individuals are responsible for the onset and recovery of their situation (“responsibility for onset,” “responsibility for recovery”). Emotionally, it is likely to show a positive and negative correlation with “anger” and “sympathy,” respectively. Additionally, because moralizing often involves a retributive interpretation, it is presumed to positively correlate with “Supernatural,” which implies rewards and punishments from supernatural forces.

Third, “Psychologizing” focuses on mental processes—such as stress and psychological burden—whose contexts are generally easily understood by others, thereby facilitating the recognition of the problem's severity. Consequently, it is predicted to correlate positively with measures of “sympathy,” “helping behavior,” and perceived “severity” toward the affected individual, and negatively with “anger.” Moreover, because the importance of psychological support is readily recognized, it is expected to correlate positively with “Family,” suggesting an orientation toward solutions provided by close others, such as family members.

Fourth, “Socializing” identifies etiology in the social environment rather than in an individual. Therefore, it is predicted to associate with the “external attribution” of causes. Social factors represent structural issues that can destabilize an individual's foundation and survival. Problems stemming from deep-rooted environmental factors are likely to be perceived as externally imposed and life-threatening, suggesting a positive correlation with “severity.” Additionally, by placing the determinants of health outside the self, it is presumed to positively correlate with “Supernatural,” “Family,” and “Professional,” which are broadly external locus types.

Fifth, “Spiritualizing” posits that spiritual or religious factors influence symptoms. Accordingly, it is predicted to correlate positively with the conceptually similar “Supernatural” dimension. Moreover, focusing on spiritual factors is akin to perceiving phenomena that are difficult to explain through physical causality; thus, it is presumed to correlate positively with “Chance”—the belief that health is dictated by luck or coincidence. Furthermore, if the spiritual framework is interpreted as a “punishment for deviating from norms,” it may facilitate attributing causes to an individual's own actions, leading to predicted positive correlations with “controllability” and “anger.” A summary of these hypotheses for verifying convergent validity is presented in Table 2.

**Table 2:** Hypothesis for validity verification.

	Explanatory Models Scale for Mental Disorders (EMS-MD)				
Variables	Medicalizing	Moralizing	Psychologizing	Socializing	Spiritualizing
Attribution of responsibility					
Outside/Inside		+		–	
Stability					
Controllability	–	+			+
Severity	+		+	+	
Responsibility for onset		+			
Responsibility for recovery		+			
Anger		+	–		+
Sympathy		–	+		
Helping behavior			+		
Japanese version of the health locus of control scales					
Supernatural		+		+	+
Internal		+			
Chance	+				+
Family			+	+	
Professional	+			+	

‘+' indicates that a positive correlation is assumed between the variables, whereas ‘–' indicates that a negative correlation is assumed.

Considering this background, this study aimed to develop the Explanatory Model Scale for Mental Disorders (EMS-MD), a measure of the multiple explanatory styles that Japanese adults use when interpreting cases in which another person exhibits symptoms of a mental disorder, and to assess its reliability and validity. In this study, the term explanatory model is used operationally, drawing on Kleinman's (1980) framework but focusing on a specific aspect of that construct. Specifically, the EMS-MD does not assess patients' subjective experiences of illness or their general beliefs about mental disorders. Rather, it assesses the context-specific explanatory styles that respondents apply to the symptoms and personal characteristics presented in a vignette representing a particular mental disorder.

## 2 Methods

### 2.1 Participants and procedure

The online survey was conducted between October and November 2025. It was administered using Google Forms via CrowdWorks Inc., (Tokyo, Japan) a Japanese crowdsourcing service. Participants were men and women aged 18 years and older. Online surveys administered through crowdsourcing platforms enable the rapid recruitment of general adult participants. However, concerns have been raised regarding inattentive responding and sample representativeness (Majima et al., 2017). To enhance data quality, participants who may not have carefully read the survey materials were excluded based on the Instructional Manipulation Check (IMC; Masuda et al., 2019) and the operation check described below. Additionally, a random subsample of 105 participants was selected to complete the same survey after a 4-week interval. Upon completion of each survey, participants received remuneration of 100 JPY.

### 2.2 Vignettes

According to Kleinman (1980), explanatory models are constructed around specific illness episodes rather than generalized beliefs about illness. Accordingly, this study used vignettes that described concrete symptoms and daily challenges rather than relying solely on disorder labels. In vignette-based surveys, respondents evaluate a specific, hypothetical situation (Hayashi, 2010). This approach was well suited to this study because it elicited case-based judgments regarding causes, meanings, and necessary support, rather than abstract beliefs about mental illness.

The study employed five vignettes representing depression (Yoshioka and Misawa, 2012), schizophrenia (Uchida et al., 2020), alcohol use disorder (Koyama et al., 2011), anorexia nervosa (Nishida et al., 2018), and social anxiety disorder (Jorm et al., 2007). The vignette for social anxiety disorder developed by Jorm et al. (2007) was translated into Japanese with the original authors' permission. Back-translation was not performed. Instead, the Japanese translation was reviewed by one clinically qualified faculty member and two graduate students in clinical psychology to ensure that it accurately reflected the clinical features of social anxiety disorder. The remaining four vignettes were used without modification, with permission from their respective original authors. Descriptions of each vignette are presented in Table 3.

**Table 3:** Vignettes used in the survey.

Depression (Yoshioka and Misawa, 2012)	Mr. A (Ms. B) is 30 years old. He (She) has been feeling unusually sad and miserable for the last few weeks. Even though he (she) is tired all the time, he (she) has trouble sleeping nearly every night. He (She) doesn't feel like eating and has lost weight. He (She) can't keep his (her) mind on work and puts off making any decisions. Even day-to-day tasks seem too much for him (her). His (Her) boss has also noticed this and is concerned about his (her) lowered productivity. Mr. A (Ms. B) feels he (she) will never be happy again and believes his (her) family would be better off without him (her). Mr. A (Ms. B) has been so desperate to escape the pain that he (she) has been thinking of ways to end his (her) life.
Schizophrenia (Uchida et al., 2020)	A is a high school student. About 6 months ago, A started feeling that classmates were making fun of him/her for no particular reason. Recently, A believes that his/her thoughts and secrets are known even to strangers, and that this is why A is being mocked. For the past month, A has occasionally been absent from school and spends more time at home. Even when alone at home, A hears voices every day saying bad things about him/her, such as “Stupid,” which causes severe distress. When trying to study, A's mind becomes confused and unable to concentrate. Almost every day, A just lies around at home doing nothing.
Alcohol use disorder (Koyama et al., 2011)	Mr. A is 45 years old. Originally, he had a low tolerance for alcohol; he would only drink when invited and would get dead drunk on just two cans of beer (350 ml each). However, about 15 years ago, he started drinking every day due to interpersonal relationship troubles. For the past 10 years, he has been drinking a whole bottle of shochu (720 ml) in a single night. For the last 2 to 3 years, the day after drinking with friends, it has been increasingly pointed out to him that he does not remember what happened while drinking. His wife has repeatedly told him to cut down on his drinking, but he cannot. When he comes home and finds no alcohol, he thinks he should make it an alcohol-free day, but he feels restless and goes out to buy some. Since nearby shops close early, he often has to go all the way to a supermarket in the next town. Moreover, when he had to work late and couldn't drink, he felt awful, started sweating, and his hands began to tremble. Drinking a beer on his way home made the sweating and tremors subside. Recently, he cannot concentrate on his work, makes many careless mistakes, and tends to fall behind. His boss often says to him half-jokingly, “Mr. A, you always seem hungover. Are you okay?” He intends to limit his drinking to an amount that won't leave him hungover the next day, but he ends up drinking from the time he gets home until late at night. Because of this, he cannot wake up the next morning and is sometimes late for work. He thinks he shouldn't continue like this, but he cannot reduce his drinking. On his days off, he drinks with alcohol close at hand right from the daytime.
Anorexia nervosa (Nishida et al., 2018)	A is a 15-year-old female student. A has always been slim, but changes in her body shape during puberty became a major source of distress. Since then, she has been thinking about nothing but losing weight. When she was 13, A joined a fitness club and started daily workouts. Through these efforts, her weight began to drop. At the same time, she started a strict dietary restriction based on rigid rules, such as not eating fatty foods, not snacking, and eating only fixed amounts of “healthy foods.” There are days when she eats absolutely nothing. This combination of diet and exercise caused her weight to drop even further. A's current weight is significantly lower than the average weight for her height and age, and her menstruation has stopped. Although she is extremely thin, A absolutely refuses to acknowledge this. She is terrified of “getting fat” and will do anything to avoid gaining weight. She also refuses to admit that she needs help. As a result, her relationship with her family has deteriorated, and her grades at school have begun to drop.
Social anxiety disorder (Jorm et al., 2007)	Mr.A is a 15-year-old living at home with his parents. Since starting his new school last year he has become even more shy than usual and has made only one friend. He would really like to make more friends but is scared that he'll do or say something embarrassing when he's around others. Although his work is OK he rarely says a word in class and becomes incredibly nervous, trembles, blushes and seems like he might vomit if he has to answer a question or speak in front of the class. At home, Mr.A is quite talkative with his family, but becomes quiet if anyone he doesn't know well comes over. He never answers the phone and he refuses to attend social gatherings. He knows his fears are unreasonable but he can't seem to control them and this really upsets him.

Regarding the survey procedure, participants were instructed to choose one of five surveys (Surveys A–E), each featuring a specific vignette: depression (A), schizophrenia (B), alcohol use disorder (C), anorexia nervosa (D), and social anxiety disorder (E). The specific disorder names and their specific natures corresponding to each survey were concealed from the participants. After completing a demographic questionnaire, participants read their assigned vignette and answered the scale items.

### 2.3 Measures

#### 2.3.1 Demographic questionnaire

Participants were asked to provide their: age, gender, religion, and highest level of education.

#### 2.3.2 Candidate items for the EMS-MD

Items were developed to represent five explanatory styles: Haslam's (2003) “Medicalizing,” “Moralizing,” and “Psychologizing,” along with “Socializing” and “Spiritualizing.” Each category included items addressing causes, factors related to symptom persistence or worsening, and improvement or coping. The initial pool comprised 66 items: 12 for “Medicalizing,” 13 for “Psychologizing,” 15 for “Moralizing,” 15 for “Socializing,” and 11 for “Spiritualizing.” To avoid assuming any particular religion or cultural tradition, “Spiritualizing” items were standardized using neutral terms such as spiritual and religious. When examples were provided, multiple examples were included to minimize bias toward specific religious terminology. Additionally, items were written to avoid disorder-specific language so that they could be applied across all five vignettes. Examples of “Medicalizing” items included “I think this condition is strongly influenced by heredity” and “I think this condition is likely to worsen if left untreated.” Examples of “Psychologizing” items included “I think prolonged stress and accumulated psychological burden triggered this condition” and “I think there was major stress before these symptoms appeared.” Examples of “Moralizing” items included “I think this condition may improve through endurance or willpower.” Examples of “Socializing” items included “I think problems in workplace or school systems and culture are related to this condition.” Examples of “Spiritualizing” items included “I think the workings of invisible beings, such as gods or spirits, are involved in this condition.” During the item development process, content validity was confirmed by two faculty members holding a certification in clinical psychology and four graduate students majoring in clinical psychology.

The instructions read: “To what extent do you think the following statements apply to A's condition? For each item, please select the one number that most closely matches your opinion. There are no ‘right' or ‘wrong' answers. Please answer as you truly feel.” Responses were rated on a 7-point Likert scale ranging from 1 (*strongly disagree*) to 7 (*strongly agree*), and total scores were calculated for each subscale.

#### 2.3.3 Attribution of responsibility process

The items developed by Kamoshida and Wakashima (2023) were used. Responses were rated on 7-point scales for the following dimensions: locus of causality (1 = *external*, 7 = *internal*), stability (1 = *unstable*, 7 = *stable*), controllability (1 = *uncontrollable*, 7 = *controllable*), severity (1 = *not severe*, 7 = *severe*), responsibility for onset (1 = *not responsible*, 7 = *responsible*), and responsibility for recovery (1 = *not responsible*, 7 = *responsible*). Regarding emotional reactions, the degree of anger was rated from 1 (*feel no anger*) to 7 (*feel anger*), and the degree of sympathy from 1 (*feel no sympathy*) to 7 (*feel sympathy*). For behavioral reactions, the degree of helping behavior was rated from 1 (*would not help*) to 7 (*would help*).

#### 2.3.4 Health Locus of Control (HLC)

The Japanese version of the HLC Scales (J-HLC; Horike, 1991) was used. This 25- item scale comprises five factors—“Supernatural,” “Internal,” “Chance,” “Family,” and “Professional”—with five items per subscale. Each item was rated on a 6-point Likert scale ranging from 1 (*strongly disagree*) to 6 (*strongly agree*). Total scores were calculated for each subscale.

#### 2.3.5 Manipulation check item

A manipulation check item was included to assess the quality of vignette recall. Immediately after presenting the vignette, the participants were asked, “Were you able to imagine A's situation? Please select the option that best applies.” Responses were rated on a 4-point scale ranging from 1 (*could not*) to 4 (*could*).

#### 2.3.6 Detection of careless responders

To identify careless responders, the IMC (Masuda et al., 2019) was employed. This task was designed to detect participants who responded without carefully reading the instructions. The IMC stated: “In internet-based surveys, it is a problem that some people lie, do not read the questions, or give careless answers. Therefore, we apologize for the imposition, but please allow us to check whether you are reading this text properly. Once you have read this text, please proceed to the next page without answering the following question (i.e., without clicking any of the options).” Participants who selected an option contrary to the instructions were considered low-quality respondents and excluded from the analysis.

### 2.4 Data analysis

To verify the scale's validity, exploratory factor analysis (EFA) and confirmatory factor analysis (CFA) were conducted using a split-sample approach. Following Iob et al. (2021), the sample's total data were randomly divided into two subsamples (training and testing). EFA was performed on the training data, while CFA was performed on the test data using IBM SPSS Amos 29. Parallel analysis was conducted using the psych package in R. All other analyses were performed using IBM SPSS Statistics 29.

Given that the EMS-MD items were generated from five theoretical categories, a 5-factor structure was initially hypothesized. However, because these categories were qualitatively generated, the factor structure was first examined using EFA. Using the training data (*n* = 350), the response distributions of the original 66 EMS-MD items were examined in terms of skewness and kurtosis. This procedure aimed to identify items showing extreme response concentration or limited variability when treating the 7-point items as approximately continuous variables and applying maximum likelihood (ML) estimation in factor analysis. Likert-type items with five to seven response categories can be treated as continuous variables in factor analysis when they do not exhibit strong floor or ceiling effects (Rhemtulla et al., 2012; Boateng et al., 2018). Additionally, Ferrando et al. (2025) noted that when response categories are concentrated at one end of the scale, observed item scores may become highly skewed and the relationship between item scores and the underlying construct may become non-linear. Since SPSS calculates the skewness and kurtosis of a normal distribution as 0, items exceeding an absolute value of 1 for either metric—indicating significant deviation from normality—were excluded. However, item deletion was guided not only by statistical criteria but also by whether the core conceptual scope of each explanatory style remained adequately represented.

Subsequently, an EFA was conducted using ML method with Promax rotation to refine the items and examine the underlying factor structure. The number of factors was determined based on the scree plot criterion, parallel analysis, and factor interpretability considered jointly. The criteria for item retention were a primary factor loading of ≥0.40, cross-loadings on other factors of < 0.30, and a difference between primary and secondary loadings of ≥0.15. The EMS-MD items were Likert-type and therefore ordinal in nature; however, all items used a 7-point Likert scale. Prior studies have shown that Likert items with a sufficient number of categories and without extreme skew can be treated as approximately continuous in factor analysis (Norman, 2010; Rhemtulla et al., 2012). Accordingly, the EMS-MD items were treated as approximately continuous, and ML estimation was used to examine their factor structure.

CFA was conducted using the test data (*n* = 578). Model fit was evaluated using the comparative fit index (CFI), root mean square error of approximation (RMSEA), and standardized root mean square residual (SRMR). The criteria for model fit were set as excellent (CFI > 0.95, RMSEA < 0.05, SRMR < 0.05) and acceptable (CFI > 0.90, RMSEA < 0.10, SRMR < 0.10) (Hoshino et al., 2005). Additionally, the Tucker–Lewis index (TLI) and the X^2^/df ratio were used as supplementary fit indices. When model fit was inadequate, modification indices (MI) were consulted. Because MI-based modifications are *post hoc* and may increase the risk of overfitting (Hermida, 2015), correlated error terms were introduced only sparingly. Specifically, correlated residuals were considered only for item pairs within the same factor that showed clear similarity in content or wording. No correlated error covariances were specified between items loading on different factors or in the absence of strong theoretical justification. As an additional analysis, multiple-group CFA was conducted to test for measurement invariance across gender and vignettes. A non-significant Δχ^2^ value, along with ΔCFI < 0.010 and ΔRMSEA < 0.015, indicated no significant change in model fit. Scale reliability was examined by calculating Cronbach's α and McDonald's ω. A Cronbach's α of ≥0.70 was considered acceptable. Test-retest reliability was assessed using a two-way random-effects intraclass correlation coefficient (ICC) for absolute agreement. ICC values were evaluated based on the following criteria: < 0.50 = poor, 0.50–0.75 = moderate, 0.75–0.90 = good, and >0.90 = excellent (Koo and Li, 2016). To examine convergent validity, correlations were computed between the EMS-MD subscale scores, responsibility attribution measures, and J-HLC subscale scores. Discriminant validity was assessed using the Fornell–Larcker criterion, that is, whether the square root of the average variance extracted (AVE) for each factor exceeded its correlations with other factors (Fornell and Larcker, 1981). Meeting this criterion indicates that each factor explains more variance in its own indicators than it shares with other factors.

### 2.5 Ethical considerations

Before starting the survey, participants were briefed on its purpose, that their participation was entirely voluntary, there would be no disadvantages for not responding, they could withdraw from the survey at any time, and how their data and personal information would be handled and protected. Thereafter, they were asked to provide their consent for participation. This study was conducted with approval from the Research Ethics Committee of the Graduate School of Education, Tohoku University (ID: 25-1-071).

## 3 Result

### 3.1 Participants

In total, 996 individuals participated in the survey. After excluding 8 careless responders (based on the IMC) and 60 participants who failed the manipulation check (answering “could not” or “could not very well”), the final analytical sample consisted of 928 participants (450 men, 467 women, 11 preferred not to answer; mean age = 38.80, SD = 9.60). Demographic characteristics for the vignette groups are presented in Table 4. Notably, 77% of the participants identified as “non-religious.”

**Table 4:** Demographic information by groups, *n* (%).

Variables	Depression	Schizophrenia	Alcohol use disorder	Anorexia nervosa	Social anxiety disorder
	(*n* = 179)	(*n* = 198)	(*n* = 186)	(*n* = 181)	(*n* = 183)
Age, mean ± SD	32.81 ± 5.93	38.66 ± 10.48	41.08 ± 10.25	41.31 ± 9.42	40.01 ± 8.50
Gender					
Male	62 (34.6)	101 (51.0)	84 (44.9)	100 (55.2)	103 (56.3)
Female	114 (63.7)	95 (48.0)	102 (54.5)	78 (43.1)	78 (42.6)
No response	3 (1.7)	2 (1.0)	1 (0.5)	3 (1.7)	2 (1.1)
Religion					
Non-religious	144 (80.4)	147 (74.2)	150 (80.2)	137 (75.7)	137 (74.9)
Buddhism	32 (17.9)	45 (22.7)	30 (16.0)	39 (21.5)	40 (21.9)
Christianity	1 (0.6)	4 (2.0)	5 (2.7)	4 (2.2)	4 (2.2)
Other	2 (1.1)	2 (1.0)	2 (1.1)	1 (0.6)	2 (1.1)
Highest level of education attained					
Elementary/junior high school	0 (0.0)	5 (2.5)	4 (2.1)	3 (1.7)	0 (0.0)
High school	36 (20.1)	33 (16.7)	34 (18.2)	40 (22.1)	40 (21.9)
Junior college/Technical college/Vocational school	35 (19.6)	37 (18.7)	31 (16.6)	20 (11.0)	30 (16.4)
University	101 (56.4)	108 (54.5)	108 (57.8)	110 (60.8)	108 (59.0)
Graduate school	7 (3.9)	15 (7.6)	10 (5.3)	8 (4.4)	5 (2.7)

SD, standard deviation.

Although no unified standard exists for required sample size in EFA, a participant-to-item ratio of 5:1 to 10:1 serves as a convenient guideline (Costello and Osborne, 2005). For the 66 items in this study, a 5:1 ratio required a minimum of 330 participants. To meet this criterion, a total of 350 participants (180 men, 166 women, 4 preferred not to answer; mean age = 39.13, SD = 9.67)—70 randomly selected from each of the five vignette groups—were used as the training data for EFA. The remaining 578 participants (270 men, 301 women, 7 preferred not to answer; mean age = 38.58, SD = 9.55) were used as the test data for CFA. Previous research suggests that EFA can yield relatively stable estimates even with moderate sample sizes, whereas CFA is more sensitive to sample size, which significantly affects fit indices and the precision of parameter estimation (Kyriazos, 2018). Consequently, to enhance the robustness of CFA, a larger proportion of the sample was allocated to it than to the EFA. Additionally, in the second survey conducted to examine test-retest reliability, all 105 participants passed the IMC and manipulation check items. Therefore, these 105 participants (comprising individuals with depression: 17, schizophrenia: 24, alcohol use disorder: 14, anorexia nervosa: 25, social anxiety disorder: 25) were included in the final analysis.

### 3.2 EFA

Regarding skewness, only one item (“I think this symptom will not easily improve in a highly stressful environment”) was less than −1, whereas five items (“I think this symptom occurs as a result of immoral actions,” “I think the workings of invisible entities such as gods or spirits are involved in this symptom,” “I think this symptom is part of the process of growth related to faith or spirituality,” “I think spiritual experiences such as forgiveness of sins, repentance, and purification lead to the alleviation of this symptom,” and “I think influences of power beyond human understanding are behind this symptom”) exceeded 1. Regarding kurtosis, two items (“I think mental burden manifests in physical reactions, which are related to this symptom,” and “I think the accumulation of small daily stresses makes this symptom more likely to occur”) exceeded 1. These items exhibited highly uneven response distributions in the Japanese general adult sample and were judged to have limited variability; they were therefore considered unsuitable as candidate items for initial scale construction. Accordingly, these eight items were excluded from subsequent analyses, and an EFA using ML method with Promax rotation was conducted on the remaining 58 items. The initial eigenvalues (percentage of variance explained) from the first factor onward were 10.19 (17.56%), 6.37 (10.99%), 3.43 (5.92%), 3.02 (5.20%), 2.43 (4.19%), 2.21 (3.81%), 1.84 (3.17%), 1.72 (2.96%), and so forth. To strengthen factor retention, a parallel analysis was conducted, which suggested a 9-factor solution. Accordingly, an EFA specifying nine factors was conducted first; however, no items meeting the retention criteria remained for the ninth factor. EFAs were then conducted for eight-, seven-, and six-factor solutions. In the eight- and seven-factor solutions, some factors retained only two items, which was considered insufficient for factor stability, as at least three items per factor are generally recommended in scale development (Carpenter, 2018). In contrast, the 6-factor solution was theoretically interpretable and consistent with the explanatory framework of this study. Accordingly, the 6-factor solution was retained based on factor stability and interpretability. Next, the analysis was rerun after excluding 20 items with factor loadings below 0.40 and three items with cross-loadings of 0.30 or higher on other factors. Consequently, a 6-factor structure consisting of 35 items was identified.

Naming of the six factors was based on their constituent items : (1) “Socializing”—“Issues with the social system underlie this symptom”; (2) “Spiritualizing”—“Consulting with religious figures or spiritual advisors helps alleviate this symptom”; (3) “Biologizing”—“Differences in brain structure and function are one of the major causes of this symptom”; (4) “Medicalizing”—Attitudes toward the medical system in general, such as “Consulting a medical institution is important for improving this symptom”; (5) “Moralizing”—“Weakness of will is related to this symptom”; (6) “Psychologizing”—Related to counseling and stress, including “Having a safe space makes it easier to alleviate this symptom.”

### 3.3 CFA

The 35 items extracted through EFA differed in the number of items retained across factors. Therefore, to avoid overrepresentation of any single explanatory style within the final scale and to balance the range and composition of subscale scores, an equal number of items was retained for each factor. In scale development, it is generally recommended that each factor include a minimum of three items, with four to five items per factor considered desirable (Carpenter, 2018). Because the factor with the fewest extracted items contained four items, four items were selected for each factor. For factors with five or more items, those with high EFA loadings and clear theoretical relevance to the corresponding explanatory style were selected. Through this procedure, 24 items were selected for the final version of the EMS-MD (Table 5). As shown in Table 5, the final items represented distinct conceptual roles of each explanatory style, suggesting that the major conceptual domains of the proposed explanatory styles remained represented in the final item set. The final 24-item Japanese version of the EMS-MD is provided in the Supplementary material.

**Table 5:** Confirmatory factor analysis results of Explanatory Models Scale for Mental Disorders (EMS-MD).

No.	Item	F1	F2	F3	F4	F5	F6	Conceptual role
*Factor 1. Socializing (α = 0.79, ω = 0.80)*								
54	Issues with the social system underlie this symptom.	0.772						Structural/systemic cause
62	Part of the responsibility for this symptom lies with society as a whole.	0.768						Social responsibility attribution
64	Social issues, such as education and labor, are related to this symptom.	0.720						Education/labor-related social determinants
29	Regional or national-level policies are effective in preventing this symptom.	0.492						Policy-level prevention and intervention
*Factor 2. Spiritualizing (α = 0.88, ω = 0.89)*								
30	Consulting with religious figures or spiritual advisors helps alleviate this symptom.		0.884					Religious/spiritual consultation
40	Religious or spiritual practices, such as prayer, meditation, or worship, are helpful in relieving this symptom.		0.794					Religious/spiritual practice
10	Religious or spiritual rituals are helpful in reducing this symptom.		0.793					Ritual-based coping
15	Visiting places considered sacred helps alleviate this symptom.		0.767					Sacred-place-based coping
*Factor 3. Biologizing (α = 0.81, ω = 0.82)*								
56	An imbalance of chemicals in the brain is one of the major causes of this symptom.			0.863				Neurochemical cause
51	Differences in brain structure and function are one of the major causes of this symptom.			0.668				Brain structure/function cause
46	An imbalance in the body, such as hormones, is related to this symptom.			0.637				Physiological/hormonal imbalance
26	Malfunctions in brain function are related to this symptom.			0.635				Brain dysfunction
*Factor 4. Medicalizing (α = 0.79, ω = 0.79)*								
16	Consulting a medical institution is important for improving this symptom.				0.819			Medical consultation
41	This symptom is likely to worsen if left untreated without medical care.				0.784			Need for medical treatment
1	This symptom can be explained as part of a medical illness.				0.644			Illness framing
6	Taking medication is effective in relieving this symptom.				0.553			Pharmacological treatment
*Factor 5. Moralizing (α = 0.76, ω = 0.76)*								
65	A lack of self-control affects this symptom.					0.781		Self-control attribution
48	This symptom can be explained as a result of one's own actions.					0.651		Personal responsibility attribution
8	Weakness of will is related to this symptom.					0.650		Weakness of will
18	This symptom is hard to improve due to character flaws.					0.453		Character flaw attribution
*Factor 6. Psychologizing (α = 0.63, ω = 0.64)*								
32	Prolonged stress and the accumulation of psychological burdens are triggers for this symptom.						0.673	Accumulated psychological stress
22	Having a safe space makes it easier to alleviate this symptom.						0.566	Safe psychological environment
28	Changing negative thoughts helps improve this symptom.						0.428	Cognitive change
2	Counseling can be expected to be effective in alleviating this symptom.						0.361	Psychological support/counseling
	F1	–						
	F2	0.361	–					
	F3	0.350	0.213	–				
	F4	0.135	−0.164	0.384	–			
	F5	0.185	0.316	0.137	−0.077	–		
	F6	0.385	−0.099	0.380	0.415	−0.023	–	
	The square roots of the AVE	0.698	0.810	0.707	0.708	0.644	0.521	

The numbers in the leftmost column indicate the item order in the EMS-MD as administered in this study. The English items are translated from the original Japanese items.

To verify the fit of this 6-factor, 24-item model, a CFA was conducted using the test data (*n* = 578). The results indicated that the initial model (Table 6, 6-factor model 1) exhibited an insufficient fit. Therefore, based on the MI, we considered specifying correlated error terms as a *post hoc* model modification. Accordingly, three error covariances (between items 51–26, 56–46, and 8–18) were specified focusing only on items within the same factor that had high MI values and shared similar content or expressions. Consequently, the modified model (Table 6, 6-factor model 2) demonstrated an acceptable fit [χ^2^(234) = 715.264, χ^2^*/df* = 3.06, CFI = 0.903, TLI = 0.885, RMSEA = 0.060 (90% CI = 0.055–0.065), SRMR = 0.066].

**Table 6:** Index of fit in the confirmatory factor analysis of Explanatory Models Scale for Mental Disorders (EMS-MD).

Model	*χ^2^*	*df*	CFI	TLI	RMSEA	90% CI RMSEA	SRMR
5-factor model	1,315.157	242	0.783	0.753	0.088	0.083–0.092	0.091
6-factor model 1	769.329	237	0.892	0.875	0.062	0.058–0.067	0.069
6-factor model 2	715.264	234	0.903	0.885	0.060	0.055–0.065	0.066

CFI, comparative fit index; TLI, Tucker-Lewis Index; RMSEA, root mean square error of approximation; SRMR, standardized root mean square residual; CI, confidence interval.

To examine the robustness of the exploratory distinction between “Biologizing” and “Medicalizing,” an additional sensitivity analysis was conducted. “Biologizing” and “Medicalizing,”—which were separated during the EFA—were combined into a single factor, and this alternative 5-factor model was compared with the 6-factor model. The 6-factor model showed better fit than the 5-factor model (Table 6, 5-factor model). To further examine the stability of this exploratory distinction, a bootstrap sensitivity analysis was conducted using the 6-factor model (Table 7). Bias-corrected 95% confidence intervals were estimated based on 2,000 bootstrap samples. The standardized factor loadings for the “Medicalizing” items ranged from 0.557 to 0.818. Similarly, the standardized factor loadings for the “Biologizing” items ranged from 0.510 to 0.823. The standardized correlation between “Biologizing” and “Medicalizing” was moderate [*r* = 0.390, 95% CI (0.268, 0.498)]. Together with the better fit of the 6-factor model compared with the 5-factor model, these results provide additional preliminary support for the stability of the exploratory distinction between “Biologizing” and “Medicalizing.”

**Table 7:** Bootstrap sensitivity analysis for the biologizing and medicalizing factors.

Factor	Item	Standardized loading/ correlation	Bias-corrected 95% CI
Medicalizing	16	0.818	[0.753, 0.874]
Medicalizing	41	0.783	[0.733, 0.830]
Medicalizing	1	0.645	[0.567, 0.717]
Medicalizing	6	0.557	[0.474, 0.637]
Biologizing	51	0.823	[0.768, 0.873]
Biologizing	26	0.805	[0.751, 0.855]
Biologizing	56	0.735	[0.660, 0.795]
Biologizing	46	0.510	[0.398, 0.611]
Biologizing ↔ Medicalizing	—	0.390	[0.268, 0.498]

Bootstrap confidence intervals are bias-corrected 95% confidence intervals based on 2,000 bootstrap samples. The final row represents the standardized correlation between the biologizing and medicalizing factors.

### 3.4 Multiple-group analysis

Measurement invariance across genders was examined (Table 8). First, regarding the model fit for each gender, the results for men were χ^2^(234) = 518.386, χ^2^*/df* = 2.22, CFI = 0.877, TLI = 0.855, RMSEA = 0.067 [90% CI = 0.059–0.075], SRMR = 0.081, and for women were χ^2^(234) = 476.378, χ^2^*/df* = 2.04, CFI = 0.904, TLI = 0.887, RMSEA = 0.059 [90% CI = 0.051–0.066], SRMR = 0.070. Although the CFI for men did not meet the criterion, the RMSEA and SRMR were within acceptable ranges. Multiple-group CFA showed that the fit of the configural invariance model was χ^2^(468) = 994.783, χ^2^*/df* = 2.13, CFI = 0.891, TLI = 0.872, RMSEA = 0.044 [90% CI = 0.041–0.048], SRMR = 0.081. Next, the metric invariance model fit was χ^2^(486) = 1,017.206, χ^2^*/df* = 2.09, CFI = 0.890, TLI = 0.875, RMSEA = 0.044 [90% CI = 0.040–0.048], SRMR = 0.081. The changes were limited to ΔCFI = 0.001 and ΔRMSEA = 0.000, indicating no significant decrease in fit [Δχ^2^(18) = 22.423, *p* = 0.214]. In contrast, the scalar invariance model fit was χ^2^(510) = 1,070.025, χ^2^*/df* = 2.10, CFI = 0.884, TLI = 0.875, RMSEA = 0.044 [90% CI = 0.040–0.048], SRMR = 0.081. Compared to the metric invariance model, although ΔCFI < 0.010 and ΔRMSEA < 0.010, the Δχ^2^ indicated a significant change [Δχ^2^(24) = 52.819, *p* = 0.001].

**Table 8:** Measurement invariance of model 2 among gender groups for Explanatory Models Scale for Mental Disorders (EMS-MD).

Group/model		Baseline						Differences				
	*χ^2^*	*df*	CFI	TLI	RMSEA	90% CI RMSEA	SRMR	Δ*χ^2^*	Δ*df*	*p*	ΔCFI	ΔRMSEA
Male	518.386	234	0.877	0.855	0.067	0.059–0.075	0.081	–	–	–	–	–
Female	476.378	234	0.904	0.887	0.059	0.051–0.066	0.070	–	–	–	–	–
Configural	994.783	468	0.891	0.872	0.044	0.041–0.048	0.081	–	–	–	–	–
Metric	1,017.206	486	0.890	0.875	0.044	0.040–0.048	0.081	22.423	18	0.214	0.001	0.000
Scalar	1,070.025	510	0.884	0.875	0.044	0.040–0.048	0.081	52.819	24	0.001	0.006	0.000

CFI, comparative fit index; TLI, Tucker-Lewis Index; RMSEA, root mean square error of approximation; SRMR, standardized root mean square residual; CI, confidence interval.

Next, measurement invariance across vignettes was examined (Table 9). First, regarding the model fit for each vignette group, although the CFI did not meet the criteria for all vignettes, the RMSEA showed good values, and the SRMR mostly indicated an acceptable fit. The results of the multiple-group CFA showed that the fit of the configural invariance model was χ^2^(1,170) = 1,939.546, χ^2^*/df* = 1.66, CFI = 0.855, TLI = 0.829, RMSEA = 0.034 [90% CI = 0.031–0.037], SRMR = 0.097. While the CFI value was low, the RMSEA and SRMR indicated an acceptable fit. Next, the fit of the metric invariance model was χ^2^(1,242) = 2,055.290, χ^2^*/df* = 1.66, CFI = 0.847, TLI = 0.829, RMSEA = 0.034 [90% CI = 0.031–0.036], SRMR = 0.102. The CFI and SRMR did not meet the criteria; although the changes were limited to ΔCFI = 0.008 and ΔRMSEA = 0.005, the Δχ^2^ showed a significant change [Δχ^2^(72) = 115.744, *p* < 0.001]. An examination of the item parameters revealed that 38% of the items varied significantly across at least two vignette conditions.

**Table 9:** Measurement invariance of model 2 among vignette groups for Explanatory Models Scale for Mental Disorders (EMS-MD).

Group/model		Baseline						Differences				
	*χ^2^*	*df*	CFI	TLI	RMSEA	90% CI RMSEA	SRMR	Δ*χ^2^*	Δ*df*	*p*	ΔCFI	ΔRMSEA
Depression	381.828	234	0.819	0.787	0.076	0.062–0.090	0.097	–	–	–	–	–
Schizophrenia	438.941	234	0.845	0.817	0.083	0.071–0.095	0.094	–	–	–	–	–
Alcohol use disorder	342.078	234	0.891	0.872	0.063	0.048–0.077	0.087	–	–	–	–	–
Anorexia nervosa	392.296	234	0.840	0.811	0.078	0.065–0.092	0.103	–	–	–	–	–
Social anxiety disorder	384.389	234	0.873	0.850	0.076	0.062–0.089	0.099	–	–	–	–	–
Configural	1,939.546	1,170	0.855	0.829	0.034	0.031–0.037	0.097	–	–	–	–	–
Metric	2,055.290	1,242	0.847	0.829	0.034	0.031–0.036	0.102	115.744	72	< 0.001	0.008	0.005

CFI, comparative fit index; TLI, Tucker-Lewis Index; RMSEA, root mean square error of approximation; SRMR, standardized root mean square residual; CI, confidence interval.

### 3.5 Reliability

Internal consistency for the EMS-MD subscales were assessed via Cronbach's α and McDonald's ω in the total sample (*n* = 928). Good internal consistency was confirmed for five factors: “Socializing” (α = 0.79, ω = 0.80), “Spiritualizing” (α = 0.88, ω = 0.89), “Biologizing” (α = 0.81, ω = 0.82), “Medicalizing” (α = 0.79, ω = 0.79), and “Moralizing” (α = 0.76, ω = 0.76). Although “Psychologizing” (α = 0.63, ω = 0.64) fell below the acceptable range, the calculated average inter-item correlation (AIIC) was 0.30.

Additionally, test-retest reliability was examined, by calculating the ICC for each EMS-MD subscale. The results were 0.85 [95% CI (0.78–0.90)] for “Socializing,” 0.83 [95% CI (0.73–0.89)] for “Spiritualizing,” 0.81 [95% CI (0.72–0.87)] for “Biologizing,” 0.83 [95% CI (0.66–0.91)] for “Medicalizing,” 0.92 [95% CI (0.89–0.95)] for “Moralizing,” and 0.88 [95% CI (0.82–0.92)] for “Psychologizing.” The above results and descriptive statistics for the EMS-MD are presented in Table 10.

**Table 10:** Descriptive statistics of Explanatory Models Scale for Mental Disorders (EMS-MD).

Subscale	Mean	SD	α	ω	ICC	90% CI ICC
Socializing	16.66	4.35	0.79	0.80	0.85	0.78–0.90
Spiritualizing	9.34	4.71	0.88	0.89	0.83	0.73–0.89
Biologizing	17.02	4.43	0.81	0.82	0.81	0.72–0.87
Medicalizing	21.58	3.91	0.79	0.79	0.83	0.66–0.91
Moralizing	13.70	4.53	0.76	0.76	0.92	0.89–0.95
Psychologizing	21.41	3.09	0.63	0.64	0.88	0.82–0.92

ICC, intra-class correlation coefficient; CI, confidence interval.

### 3.6 Convergent validity and discriminant validity

The convergent validity of the EMS-MD was examined through correlation analyses between its subscale scores, the attribution of responsibility process scores, and the J-HLC subscale scores (Table 11). Although trivial correlations may reach statistical significance in large sample sizes, the main text focuses on the correlations most relevant to the hypotheses.

**Table 11:** Correlations between Explanatory Models Scale for Mental Disorders (EMS-MD) and other scales.

	EMS-MD					
Variables	Socializing	Spiritualizing	Biologizing	Medicalizing	Moralizing	Psychologizing
Attribution of responsibility						
Outside/Inside	−0.283^***^	0.044	0.028	−0.104^**^	0.326^***^	−0.090^**^
Stability	0.092^**^	−0.146^***^	0.081^*^	0.239^***^	−0.017	0.153^***^
Controllability	−0.098^**^	0.150^***^	−0.071^*^	−0.413^***^	0.292^***^	−0.098^**^
Severity	0.221^***^	−0.101^**^	0.170^***^	0.424^***^	−0.160^***^	0.274^***^
Responsibility for onset	−0.057	0.132^***^	−0.037	−0.087^**^	0.612^***^	−0.125^***^
Responsibility for recovery	−0.040	0.041	−0.026	−0.138^***^	0.375^***^	−0.020
Anger	0.056	0.207^***^	−0.033	−0.084^*^	0.396^***^	−0.268^***^
Sympathy	0.182^***^	−0.023	0.131^***^	0.149^***^	−0.254^***^	0.299^***^
Helping behavior	0.190^***^	0.097^**^	0.080^*^	0.107^**^	−0.149^***^	0.261^***^
Japanese version of the health locus of control scales						
Supernatural	0.202^***^	0.580^***^	0.105^**^	−0.184^***^	0.308^***^	−0.122^***^
Internal	0.061	−0.052	0.078^*^	0.179^***^	0.240^***^	0.333^***^
Chance	0.228^***^	0.265^***^	0.198^***^	−0.046	0.149^***^	−0.081^*^
Family	0.295^***^	0.051	0.156^***^	0.249^***^	0.020	0.448^***^
Professional	0.124^***^	−0.021	0.183^***^	0.263^***^	0.092^**^	0.157^***^

^*^p < 0.05, ^**^p < 0.01, ^***^p < 0.001.

As shown in Table 11, “Spiritualizing” was strongly and positively correlated with “Supernatural” (*r* = 0.580, *p* < 0.001). “Moralizing” was positively correlated with “Responsibility for Onset” (*r* = 0.612, *p* < 0.001), “Anger” (*r* = 0.396, *p* < 0.001), “Responsibility for Recovery” (*r* = 0.375, *p* < 0.001), and “Outside/Inside” (*r* = 0.326, *p* < 0.001), and negatively correlated with “Sympathy” (*r* = −0.254, *p* < 0.001). “Medicalizing” was positively correlated with “Severity” (*r* = 0.424, *p* < 0.001) and “Professional” (*r* = 0.263, *p* < 0.001), and negatively correlated with “Controllability” (*r* = −0.413, *p* < 0.001). In contrast, “Biologizing” was positively correlated with “Chance” (*r* = 0.198, *p* < 0.001) and “Professional” (*r* = 0.183, *p* < 0.001). The remaining correlations are listed in Table 11.

Discriminant validity was assessed using the Fornell–Larcker criterion. For all factors, the square root of the AVE exceeded the absolute values of the inter-factor correlations. Notably, the correlation between “Biologizing” and “Medicalizing” was *r* = 0.384, whereas the square roots of their AVEs were 0.707 and 0.708, respectively.

## 4 Discussion

This study developed the EMS-MD to assess how Japanese adults understand mental disorders presented in vignettes and examined its reliability and validity. The main findings are as follows. First, the EMS-MD was interpretable as a 6-factor structure comprising “Socializing,” “Spiritualizing,” “Biologizing,” “Medicalizing,” “Moralizing,” and “Psychologizing.” Second, items initially assumed to belong to the broader category of “Medicalizing” were separated into “Biologizing” and “Medicalizing.” Third, although several subscales showed acceptable evidence of reliability and validity, limitations remained in model fit, the internal consistency of “Psychologizing,” and measurement invariance across vignettes. The findings are discussed below.

### 4.1 The multidimensionality of explanatory styles

The EFA and CFA supported a 6-factor structure for the EMS-MD. This suggests that Japanese adults conceptualize mental disorders not only in medical and psychological terms but also through social, spiritual, and moral frameworks.

“Socializing” refers to explanations of the causes and course of mental disorders in terms of social environments and structures rather than individual traits. The risk and course of mental disorders have been linked to structural factors such as poverty, unemployment, housing insecurity, and discrimination (Kirkbride et al., 2024), and lay accounts similarly reference such factors (Choudhry et al., 2016). The emergence of “Socializing” as an independent factor therefore suggests that lay understandings of mental disorders are not reducible to intrapersonal causes.

“Spiritualizing” refers to the interpretation of symptoms through the lens of spiritual influences, religious practices, or supernatural forces. Explanatory model research has repeatedly documented spiritual framings of mental disorders across cultures (Weiss, 1997; Lloyd et al., 1998; McCabe and Priebe, 2004; Bhikha et al., 2012). In this study's sample, 77% of participants reported having “no religion,” yet “Spiritualizing” still emerged as an independent factor. This suggests that spiritualizing explanations in Japan may arise not only from formal religious affiliation but also from folk beliefs and customary religiosity. Historically, mental distress in Japan has been interpreted as a supernatural phenomenon, such as kitsune-tsuki (fox possession), and addressed through prayer and folk practices (Eguchi, 1988). It has also been noted that many Japanese people identify as “non-religious” while still engaging in customary religious practices in everyday life (Ishii, 1994). This study's finding is consistent with Dumke et al.'s (2023) proposed biopsychosocial-spiritual model.

“Moralizing” refers to explanations of mental disorders as stemming from weakness, character flaws, or moral responsibility. This factor exhibited a strong association with measures of attribution of responsibility and may therefore be relevant for future research concerning stigma and social distance. By contrast, “Psychologizing” refers to understanding symptoms in terms of stress, negative thinking, counseling, and supportive environments, and was broadly consistent with Haslam's (2003) conceptualization.

### 4.2 The distinction between “Biologizing” and “Medicalizing”

Items initially assumed to represent the broader category of “Medicalizing” emerged as two distinct factors: “Biologizing” and “Medicalizing.” Because this distinction was not hypothesized a priori, it should be interpreted as exploratory and requiring further validation.

However, this distinction holds theoretical significance. “Biologizing” attributes symptoms to biological mechanisms such as brain function and neurotransmitter activity and can be linked to broader biologized or geneticized views of human problems (Lippman, 1991). In contrast, “Medicalizing” frames symptoms as problems that need to be addressed by medical institutions, professionals, and medication. In sociology, medicalization refers to the institutional process by which conditions are defined as diseases and brought under medical jurisdiction (Conrad and Bergey, 2015). Although the present “Medicalizing” factor does not directly capture this process, it reflects an individual-level tendency to view a condition as appropriately managed within a medical framework, regardless of whether its cause is understood biologically.

The findings empirically support this distinction. “Biologizing” and “Medicalizing” were distinguishable by the Fornell–Larcker criterion, and their associations with external variables differed. “Medicalizing” demonstrated stronger associations with “Severity” and “Professional,” and a moderate-to-large negative association with “Controllability.” In contrast, “Biologizing” demonstrated weaker positive correlations with “Severity,” “Chance,” and “Professional,” and no meaningful association with “Controllability.” This pattern suggests that “Biologizing” primarily reflects causal attribution to biological processes, whereas “Medicalizing” more strongly reflects perceptions of severity, reduced controllability, and the need for professional intervention.

Prior research similarly suggests that biological explanations do not necessarily reduce stigma and may be associated with perceived danger and social distance (Kvaale et al., 2013), and that attitudes vary depending on perceived treatability (Lebowitz and Ahn, 2012). Therefore, distinguishing “Biologizing” from “Medicalizing” may help clarify how explanatory models relate to stigma and attribution of responsibility. However, given the exploratory nature of this finding, it should be tested in independent and cross-cultural samples.

### 4.3 Support for the factor structure and limitations of model fit

The revised 6-factor model showed improved fit compared with the five-factor model, and the RMSEA, SRMR, and χ^2^/df values were within acceptable ranges. However, the CFI (0.903) and TLI (0.885) did not meet the stricter recommended thresholds proposed by Hu and Bentler (1999). Therefore, although the 6-factor structure is supported, it cannot be regarded as fully established.

Additionally, three correlated residuals were specified based on modification indices, restricted to item pairs within the same factor showing similar content or wording. These involved a pair of items on brain function within “Biologizing,” a pair concerning bodily or physiological balance within “Biologizing,” and a pair concerning “weak will” and “character flaws” within “Moralizing.” These correlated residuals likely reflect local overlaps in content or phrasing. However, because these modifications were introduced *post hoc*, they may also indicate redundancies in item wording. Future research should therefore refine the item pool and test the reproducibility of this 6-factor structure.

### 4.4 Measurement invariance

Across gender, increases in model restrictions produced only small declines in fit, with ΔCFI and ΔRMSEA within acceptable thresholds. Although χ^2^ difference tests were significant for scalar models, ΔCFI and ΔRMSEA remained acceptable. Therefore, the EMS-MD appears to have a broadly similar structure for men and women, and comparison of subscale scores across sexes may be possible. However, as the CFI and TLI were unsatisfactory for all models, including the configural model, conclusions regarding gender differences should be treated as exploratory.

Invariance across vignettes was less supported. Although RMSEA and SRMR were generally acceptable, CFI and TLI values were consistently below recommended cutoffs in vignette-specific models. This suggests that while the general factor structure may be partially stable across contexts, full structural equivalence was not achieved. Moreover, approximately 38% of items showed meaningful loading differences across vignette conditions. Therefore, EMS-MD scores should not be assumed to function equivalently across disorder contexts, limiting comparisons of latent means and structural relations across vignettes.

These findings suggest that explanatory models should be understood in terms of both stability and context dependence. While Haslam's (2003) framework describes relatively general explanatory styles, Kleinman's (1980) framework emphasizes the understanding constructed in relation to specific illness episodes. Accordingly, the EMS-MD factors may represent a repertoire of explanatory styles available to lay adults; however, the extent to which each style is used and the degree to which individual items reflect a factor may vary across disorder contexts. For instance, the item “I think this symptom is related to weak will” may reflect insufficient effort or self-responsibility in a depression vignette, but a moral judgment about failure to control drinking in an alcohol dependence vignette. Both interpretations fall within “Moralizing,” but the extent to which the item reflects that factor may differ by vignette. The limited support for invariance across the vignettes is consistent with this contextual dependence.

Overall, the EMS-MD should be understood less as a measure of fixed beliefs about mental disorders in general than as a measure of how explanatory styles are organized within specific disorder contexts. Currently, it is better described as the overall profile of explanatory styles within each vignette or within sets of similar vignettes than as a direct comparison of mean scores or correlational patterns across vignettes.

### 4.5 Evidence for reliability and validity

Cronbach's α and McDonald's ω indicated generally good internal consistency across five factors, with the exception of “Psychologizing.” Although the final item set retained the major conceptual domains of the proposed explanatory styles, “Psychologizing” showed relatively low internal consistency. This suggests that preserving conceptual coverage does not necessarily imply that all subscales represent equally coherent latent constructs. “Psychologizing” included multiple aspects of psychological understanding and support, such as a safe environment, negative thinking, counseling, and prolonged stress. The small number of items may also have contributed, given that α is sensitive to item number (Tavakol and Dennick, 2011). However, the AIIC was 0.30, which falls within the recommended range (Clark and Watson, 1995; Bohrer et al., 2015), suggesting that item interrelatedness was not unduly weak. Nonetheless, the conceptual scope of “Psychologizing” warrants further clarification, and its item pool should be either refined or expanded. The test-retest reliability was good for all six factors, with ICCs ranging from 0.81 to 0.92.

Convergent validity was examined through correlations with the attribution of responsibility process measures and the J-HLC subscales. Given the large sample size, validity was evaluated mainly in terms of effect size and theoretical consistency, rather than statistical significance alone. Overall, the findings are broadly consistent with the hypotheses in Table 2. “Spiritualizing” showed a strong positive correlation with “Supernatural,” whereas “Moralizing” showed moderate or stronger correlations with “Responsibility for Onset,” “Responsibility for Recovery,” “Anger,” and “Outside/Inside.” “Socializing” was associated with “Family” and “Professional,” and “Psychologizing” with “Severity,” “Sympathy,” and “Helping Behavior.” Additionally, “Medicalizing” showed moderate positive correlations with “Severity” and “Professional,” and a moderate-to-large negative correlation with “Controllability,” suggesting a tendency to view symptoms as serious, beyond personal control, and in need of professional intervention. “Biologizing” showed small-to-moderate positive correlations with “Severity,” “Chance,” and “Professional,” suggesting that biologically framed problems may be seen as partly incidental and beyond personal effort alone. The Fornell–Larcker results provided preliminary evidence for discriminant validity: for all factors, the square root of the AVE exceeded the absolute value of its correlations with other factors, including the conceptually close pair of “Biologizing” and “Medicalizing.”

Overall, these findings provide preliminary support for the validity of the EMS-MD. However, as most correlations were small to moderate, further validation using independent samples and refined item content is required.

### 4.6 Limitations and future directions

This study had several limitations. First, the sample consisted of Japanese adults from the general population. It remains unclear whether the factor structure generalizes to individuals with mental disorders or their family members, who may hold different explanatory models based on lived experience. Future research should examine clinical and caregiver samples and test group differences.

Second, data were collected through crowdsourcing (CrowdWorks). Although this approach enables efficient recruitment, concerns remain regarding representativeness and inattentive responding. In this study, inattentive responses were screened using instructional manipulation and operation checks; however, the sample cannot be considered representative of the entire Japanese adult population. Therefore, replication using diverse sampling methods is required.

Third, the vignette method itself has limitations. Participants were asked to evaluate hypothetical cases; however, explanatory styles may differ in reality when the subject is a close family member or friend. Future research should test more ecologically valid designs, such as comparisons based on prior contact experience or procedures asking respondents to think of a specific acquaintance.

Fourth, the translation process for the social anxiety disorder vignette was limited. The vignette from Jorm et al. (2007) was translated into Japanese; however, no back-translation was conducted. Consequently, semantic equivalence with the original vignette may not have been fully achieved. Future research should adopt translation procedures, including back-translation, to establish semantic equivalence more rigorously.

Fifth, the cultural scope of validity remains limited. The EMS-MD was developed within the Japanese cultural context, and its factor structure may represent culture-specific beliefs. As explanatory models are fundamentally embedded within cultural and societal contexts (Kleinman, 1980), it is unclear whether the scale can be applied to other cultural settings with different religious views or medical systems. Future research should develop versions in other languages and test cross-cultural measurement invariance to clarify both the universal and culture-specific aspects of explanatory models.

Sixth, this study may have been affected by response bias. All data were collected through self-report questionnaires, and the potential influence of social desirability could not be excluded. Specifically, responses to “Moralizing” items may have been suppressed because the perception of mental illness as indicative of weak will or character flaws may be considered socially inappropriate. To mitigate such bias, future studies should integrate self-report measures with implicit indicators and qualitative approaches such as interviews.

Seventh, this study treated 7-point Likert-type items as approximately continuous variables and used ML estimation in the EFA and CFA. Although this approach is generally acceptable when Likert-type items have several response categories, some candidate items showed skewness or kurtosis values close to the exclusion criterion. Therefore, future research should examine the robustness of the EMS-MD factor structure using ordinal-data methods, such as polychoric correlations and WLSMV or robust estimation.

## 5 Conclusion

This study developed the EMS-MD to assess the explanatory styles used by Japanese adults when interpreting mental disorders presented in vignettes and examined its reliability and validity. The findings indicated that the EMS-MD is interpretable as a 6-factor structure comprising “Socializing,” “Spiritualizing,” “Biologizing,” “Medicalizing,” “Moralizing,” and “Psychologizing.” Additionally, most subscales demonstrated acceptable internal consistency and test-retest reliability, and their associations with external variables were broadly consistent with theoretical expectations. These findings provide preliminary evidence supporting the interpretation of EMS-MD scores as indicators of explanatory styles for mental disorders among Japanese adults.

Simultaneously, limitations remained in CFA model fit, the internal consistency of “Psychologizing,” and measurement invariance across vignettes. Therefore, the results do not indicate that the EMS-MD is a fully established instrument. In particular, the low internal consistency of “Psychologizing” suggests that this subscale may not yet represent a sufficiently coherent latent construct. Further evidence is required through refinement of item content, especially for the “Psychologizing” subscale, examination of the EMS-MD's applicability to clinical samples, and tests of measurement invariance across cultural contexts.

## Data Availability

The dataset presented in this study is publicly available in Figshare at https://doi.org/10.6084/m9.figshare.31796791.
