# Supplementary material for: Developing a comprehensive scale for measuring explanatory models of mental disorders among Japanese adults
Source: Front Psychol. 2026 Jul 29;17:1834088. doi: 10.3389/fpsyg.2026.1834088 (PMC13462051; doi:10.3389/fpsyg.2026.1834088)
Supplement: Supplementary file 1 [file Presentation_1.pdf]

# Explanatory Models Scale for Mental Disorders (EMS-MD)

A さんの状態について、以下の各文がどの程度あてはまると思いますか。各文章で、あなたの考えに最も近い数字を選んでください。**望ましい回答や正解はありません。感じたままにお答えください**。

| | | 全くそう思わない | そう思わない | あまりそう思わない | どちらともいえない | ややそう思う | そう思う | 非常にそう思う |
|---|---|---|---|---|---|---|---|---|
| 1 | この症状は、医学的な病気の一部として説明できると思う。 | 1 | 2 | 3 | 4 | 5 | 6 | 7 |
| 2 | カウンセリングには、この症状を軽くする効果が見込めると思う。 | 1 | 2 | 3 | 4 | 5 | 6 | 7 |
| 3 | 服薬は、この症状を和らげるのに有効だと思う。 | 1 | 2 | 3 | 4 | 5 | 6 | 7 |
| 4 | 意志の弱さが、この症状と関係していると思う。 | 1 | 2 | 3 | 4 | 5 | 6 | 7 |
| 5 | 宗教的・霊的な儀礼は、この症状の軽減に役立つと思う。 | 1 | 2 | 3 | 4 | 5 | 6 | 7 |
| 6 | 神聖だと感じる場所を訪れることは、この症状を和らげる助けになると思う。 | 1 | 2 | 3 | 4 | 5 | 6 | 7 |
| 7 | 医療機関で相談することは、この症状の改善のために重要だと思う。 | 1 | 2 | 3 | 4 | 5 | 6 | 7 |
| 8 | 性格上の欠点があるために、この症状は良くなりにくいと思う。 | 1 | 2 | 3 | 4 | 5 | 6 | 7 |
| 9 | 安心できる場があると、この症状は和らぎやすいと思う。 | 1 | 2 | 3 | 4 | 5 | 6 | 7 |
| 10 | 脳の機能の不具合が、この症状に関係していると思う。 | 1 | 2 | 3 | 4 | 5 | 6 | 7 |
| 11 | ネガティブな考えを改めることは、この症状の改善を後押しすると思う。 | 1 | 2 | 3 | 4 | 5 | 6 | 7 |
| 12 | この症状の予防には、地域や国家レベルの施策が有効だと思う。 | 1 | 2 | 3 | 4 | 5 | 6 | 7 |

| | | | | | | | | |
|---|---|---|---|---|---|---|---|---|
| 13 | 宗教者や霊的な助言を行う人への相談は、この症状を軽くする助けになると思う。 | 1 | 2 | 3 | 4 | 5 | 6 | 7 |
| 14 | 長引くストレスや心理的な負担の蓄積が、この症状のきっかけになっていると思う。 | 1 | 2 | 3 | 4 | 5 | 6 | 7 |
| 15 | 祈り・瞑想・礼拝などの宗教的・霊的な実践は、この症状を和らげるのに役立つと思う。 | 1 | 2 | 3 | 4 | 5 | 6 | 7 |
| 16 | 医療を受けずに放置すると、この症状は悪化しやすいと思う。 | 1 | 2 | 3 | 4 | 5 | 6 | 7 |
| 17 | この症状には、ホルモンなどの体内のバランスの乱れが関連していると思う。 | 1 | 2 | 3 | 4 | 5 | 6 | 7 |
| 18 | この症状は、自身の行いの結果として説明できると思う。 | 1 | 2 | 3 | 4 | 5 | 6 | 7 |
| 19 | 脳の構造や働きの違いが、この症状の大きな要因の一つだと思う。 | 1 | 2 | 3 | 4 | 5 | 6 | 7 |
| 20 | この症状の背景には、社会の仕組みの問題があると思う。 | 1 | 2 | 3 | 4 | 5 | 6 | 7 |
| 21 | 脳内の化学物質のバランスの乱れが、この症状の大きな要因の一つだと思う。 | 1 | 2 | 3 | 4 | 5 | 6 | 7 |
| 22 | この症状に対する責任の一部は、社会全体にもあると思う。 | 1 | 2 | 3 | 4 | 5 | 6 | 7 |
| 23 | この症状には、教育や労働などの社会問題が関連していると思う。 | 1 | 2 | 3 | 4 | 5 | 6 | 7 |
| 24 | 自己管理の不足が、この症状に影響していると思う。 | 1 | 2 | 3 | 4 | 5 | 6 | 7 |
